# Students’ perspective of bedside teaching: A qualitative study

**DOI:** 10.12669/pjms.322.9194

**Published:** 2016

**Authors:** Farhan Khashim Al-Swailmi, Ishtiaq Ali Khan, Yasir Mehmood, Shehab Ahmed Al-Enazi, Majed Alrowaili, Madallah Mashaan Al-Enazi

**Affiliations:** 1 Farhan Khashim Al-Swailmi, MD. Assistant Professor, Department of Ophthalmology, Faculty of Medicine Northern Border University Arar, Kingdom of Saudi Arabia; 2 Ishtiaq Ali khan, FCPS. Associate Professor, Department of Surgery, Frontier Medical College Abbottabad, Pakistan; 3 Yasir Mehmood, FCPS. Assistant Professor of Surgery, Department of Surgery, Faculty of Medicine Northern Border University Arar, Kingdom of Saudi Arabia; 4 Shehab Ahmed Al-Enazi, MD. Assistant Professor, Department of Pediatrics, Faculty of Medicine Northern Border University Arar, Kingdom of Saudi Arabia; 5 Majed Alrowaili, MD. Assistant Professor, Department of Orthopedics, Faculty of Medicine Northern Border University Arar, Kingdom of Saudi Arabia; 6 Madallah Mashaan Al-Enazi, MD. Assistant Professor, Department of Medicine, Faculty of Medicine Northern Border University Arar, Kingdom of Saudi Arabia

**Keywords:** Bedside Teaching, Faculty development, Students

## Abstract

**Objectives:**

To determine students’ perception of bedside teaching, to find out barriers in its effective implementation and to suggest strategies to make it an effective learning tool.

**Methods:**

This study was conducted in Faculty of Medicine, Northern Border University Arar, Kingdom of Saudi Arabia between November 2013 and January 2014. The study design was qualitative inductive thematic analysis using transcripts from audio-recorded focus group discussions. Four focused group discussions with medical students of 4^th^ and 5^th^ year MBBS were conducted. Each 40 to 50 minutes discussion session was audio taped and transcribed verbatim. Thematic analysis extracted key themes pertaining to objectives of the study.

**Results:**

A total 75 students of 4^th^ and 5^th^ year MBBS took part in the study, 48 were female and 27 of them were male. Students believed that bedside teaching is valuable for learning essential clinical skills. They described many barriers in its effective implementation: uncooperative and less number of patients and faculty attitude. Our students suggested various strategies to address these barriers: promotion of awareness among general public about students’ learning and its benefits, free medical treatment for expatriates and building of university hospital.

**Conclusion:**

Bedside teaching is an important learning tool. Its utility can be enhanced by orienting local patients’ attitude towards importance of students’ learning, by providing free medical treatment to expatriates and by including bedside teaching in faculty development programs.

## INTRODUCTION

Bedside teaching (BST) is a specialized form of small group teaching that takes place in the presence of the patient.^1^ Traditionally, BST has always been seen as a primary teaching modality in which most aspects of clinical practice can be demonstrated and trained.^2^ It remains compatible with more recently defined learning theories such as, contextual learning, where the learning of knowledge is dependent on the context in which it is learned.^3^ Although it is known to enhance student’s learning experience and improves patient care, the use of this type of teaching is unfortunately in steady decline due to multiple responsibilities of faculty members and emerging learning instructions like seminars and conferences.^1^ Role of BST in acquisition of clinical skills, communication skills and professionalism cannot be over emphasized but this happens in haphazard manner as students proceed through different clinical units.

Some studies have explored BST only from perspective of teachers but students’ point of view has not been adequately addressed.^4^ Students are important stakeholders in learning. Therefore, appreciating students’ attitudes, perception of problems and suggestions to improve BST must not be ignored.

The objectives of this study were to explore students’ perception about BST, their difficulties in clinical learning and their point of view to make BST an effective learning tool.

## METHODS

The study was conducted in Faculty of Medicine, Northern Border University Arar, Kingdom of Saudi Arabia between November 2013 and January 2014. The study design was qualitative inductive thematic analysis using transcripts from audio-recorded focus group discussions. Four separate focused group discussions with 4^th^ and 5^th^ year MBBS students were planned to collect in- depth data. Two groups of students, one female and one male, from each academic year i.e., 4^th^ and 5^th^ year were included. This sample selection was purposeful because these students were experiencing BST. Objectives of the study were explained to the students and verbal consent was taken for participation in the study. Each focus group discussion session was conducted for 40-50 minutes. Open-ended questions were used to explore students’ views on bedside teaching. Facilitator asked questions and participants were encouraged to talk freely about their experience of BST. One researcher (Ishtiaq Ali Khan) asked for clarification and further elaboration of the students’ responses where needed for saturation of data. Ethical approval of the study was sought from the institutional review board of the Faculty of Medicine.

### Statistical Analysis

Students’ answers and mutual discussions were audio taped and transcribed verbatim for content analysis.^5^ A thematic analysis extracted key themes pertaining to objectives of the study by grouping words, phrases and statements of similar meanings into categories. The final coding and themes were approved by all authors through consensus.

## RESULTS

A total 75 students took part in study, 48 were female and 27 of them were male. Female students were consisting of 24 in each group and there were 13 male students in one group and 14 were in other one. One focused group discussion session of 40-50 minutes was conducted with each group of students. The following themes emerged from these discussions.

### Bedside teaching and learning

Students believe that they learn history taking, physical examination, communication skills, case discussion, how to deal with patients, cooperation with each other and application of theoretical knowledge.

### Quantity and quality of bedside teaching

The average time for bedside teaching was from 8 to 12 hours per week which did not satisfy needs of the students: “…Not sufficient, we need more time to learn.” students observed that the time allocated for bedside teaching is not utilized properly due to multiple reasons. Conflicts with the hospital administration, less number of patients, uncooperative patients and travelling distance between hospital and university were the common reasons.

Students suggested that other areas of patients’ location like emergency and outpatient department should be explored in addition to ward. They also emphasized role of teacher to utilize time properly: “doctor (teacher) must be with his batch” … “We are mostly alone with the patient.”

### Role of teacher in bedside teaching

Difference of opinion on a topic among tutors was confusing for the students: “…yes and we feel problem sometimes… on same knowledge, different opinions… which confuses us…”

“I am student I want information from one book.” (4^th^ year female) Few students also appreciated difference of opinion as they get variety of information. Most of the students requested that all teachers should teach us from one source: “teachers should agree on one source for students.” (4^th^ year male) Students suggested some teaching workshop for the teachers.

Students also stressed on role modeling and advance planning by the teacher: “teach us from beginning how to take a good history.” Students pointed out that some teacher produce phobia about clinical examination.

Some students also demanded university hospital because non university hospital has staff from ministry of health and students said that some doctors in these hospitals refused to teach them. Moreover, in university hospital patients know that this is a university hospital and students will also examine them. Therefore, patients are expected to cooperate with students.

### Barriers in bedside teaching and their solutions

Insufficient number of patients was stated by the students as the main barrier to effective bedside teaching. Students also communicated that even if patients are present in the ward they are mostly post-operative cases where we have no opportunity to pick up pre-operative examination findings. Most of the patients in hospitals are Saudi natives which has positive and negative effects on bedside teaching as reported by the students. Students felt that due to cultural and religious reasons native female patients are non-cooperative and generally all native patients are less cooperative than expatriates but some students highlighted that it is easy to deal with native patients than expatriate patients due to cultural and linguistic similarities. Students noted that patients are less in number because they go for treatment to big cities like Riyadh or Dammam or they proceed abroad. Some students also pointed out that paramedic staff of the hospital create hurdles for them. The paramedic staff tells patients that medical students are learning on them which make patients uncooperative.

Students strongly realized the need of free treatment for all patients to improve BST. “…Only and the best solution is to build university hospital and free treatment for patients of all nationalities to improve BST.” (5^th^ year female) To facilitate BST students suggested that general public should be made aware of medical colleges and their purpose towards humanity.

Students also suggested that to decrease patient discomfort, students should resume case discussion in another room after taking history and performing examination: “…decrease the time students spend at bedside near patient, sometime students stand at bedside for hours which is terrible for the patient…”

## DISCUSSION

Our students recognized and appreciated role of BST in learning history taking, clinical examination and communication skills which are consistent with findings of other such studies conducted on BST.^4^,^6^

Literature reviews indicate that actual teaching at the bedside has declined from 75% in the 1960s to less than 8-19% recently.^1^,^7^ Along with this decrease; a decline in overall clinical skills among trainees and junior faculty members has been observed.^8^,^9^

Inadequate time for BST is frequently described as a major barrier for BST. This is of some concern when one considers the findings reported by Nair et al. Only 48% of learners reported that they had been given enough bedside teaching during their undergraduate training, while 100% thought that bedside learning was the most effective way of learning clinical skills.^10^ His findings are very consistent with our results.

Patient plays a pivotal role in success of BST but they may become unwilling to participate because of pain, anxiety, embarrassment, or “student fatigue,” where they are often the subject of multiple clinical examinations if they possess a rare or significant clinical sign. Moreover, they may be unavailable due to procedures, consultations or imaging.^4^

Nair et al.reported that a majority of patients enjoyed and benefited from bedside teaching by understanding their own problems contrary to the belief that patients are reluctant to consent for be the subjects of teaching and learning.^11^

There are other significance evidences in the literature which shows that patients often enjoy being the subject of BST.^12^,^13^ These findings are contrary to observations of our students. Our students faced uncooperative patients which may be due cultural and geographical differences among patients across the globe.

There has been a paradigm shift in clinical diagnosis. There is an increasing dependence on sophisticated technology and laboratory tests rather than clinical examination skills fostered at the bedside. This philosophy has resulted in a partial shift of ward rounds from the bedside to the conference room.^14^ Therefore our students suggested exploration of other areas of BST like emergency ward and out patients department which shows commitment of students toward their clinical learning.

The patient’s bedside appears to be one of the most challenging settings for clinical teachers. Some common sense strategies combined with faculty development programs at individual institutions can overcome teaching deficiencies of clinicians. However, only a minority of academic medical centers offer ongoing faculty development in teaching skills.^15^ Teachers need to familiarize themselves with the clinical curriculum, attempt to diagnose different learner levels and improve their own clinical skills. An important part of clinical teaching is the development of the professional role in the students. A role model teaches primarily by example, helping to shape the professional identity of medical students in preparation for their entry into the workplace as doctors.^16^

It is encouraging that our students felt the need for improvement in clinical teaching as they suggested faculty developing program to improve bedside teaching and stressed on role modeling by clinical teachers to improve bedside teaching.

Although most of the students thought that expatriate patients are more cooperative but some students rightly pointed out that though Saudi native patients are less cooperative but it is easy to build rapport with them due to linguistic and cultural similarities. The role of native language and culture in learning cannot be overemphasized. If a secondary language is used, a more limited, and possibly inaccurate, history can result in misdiagnosis.

People of diverse cultural backgrounds often make different attributions of illness, health, disease, symptoms and treatment. Cultural differences in health attributions have major implications for medical professionals because over time, attributions play an essential role in the formation of beliefs concerning health and illness.^17^

Our students suggested strategies to improve BST such as creating awareness among patients which are consistent with other studies conducted on the same issue.^4^ It was highly appreciable that students recognized the problem of expatriate patients in Saudi Arabia and suggested free medical treatment for them so that deficiency of patients for BST can be overcome. Our students realized patient’s discomfort by suggesting that after taking history and performing examination on patient, rest of the case discussion should be done in another room. A qualitative study^18^ reported that paying attention to patient’s expectations and needs results in patient’s satisfaction and decrease in hospital stay.

The importance of advance planning cannot be overemphasized and has been realized by our students. Adequate preparation is essential responsibility of a tutor to meet the goals of bedside teaching.^9^

It is interesting to find that our students recognized the value of support and encouragement to enhance learning instead of creating phobia by the teacher. Groenlund C and Handal B^19^ reported that students portray an effective teacher as someone who gives constructive feedback, who develops rapport with the students, approachable and links theory to practice. Students in our study also realized importance of feedback. Our students valued the teacher who guides them in selection of course study resource.

Student is an important stakeholder in learning but so far very few studies have explored the students’ opinion on bedside teaching. Therefore, further exploration of students’ view is direly needed to revive and improve BST.

## CONCLUSION

Bedside teaching is an important learning tool. Its utility can be enhanced by creating awareness among patients and their attendants regarding teaching and learning of medical students and its role in health care. Free of cost or economical medical treatment for expatriates may also increase the number of patients available in hospital for BST. Faculty development programs will play pivotal role to prove BST as an effective learning tool.
